# The value of multimodal MRI in the clinical grading of acute cerebral infarction

**DOI:** 10.3389/fnins.2025.1604551

**Published:** 2025-06-20

**Authors:** Haiyan Gui, Jingjing Zhang, Jianxiu Lian, Ningdi Yang, Hongwei Li

**Affiliations:** ^1^Department of Radiology, The Fourth Hospital of Harbin, Harbin, China; ^2^Clinical and Technical Support, Philips Healthcare, Beijing, China

**Keywords:** arterial spin label, magnetic resonance imaging, acute ischemic cerebral infarction, cerebrovascular circulation, cerebral perfusion

## Abstract

**Introduction:**

Acute ischemic stroke (AIS) is one of the prevalent types of stroke, characterized by high mortality and disability rates. For reperfusion therapy in acute cerebral infarction, early and accurate identification of ischemic penumbra, collateral circulation, and clinical grading is crucial for clinicians in devising effective treatment strategies and assessing prognosis.

**Methods:**

Patients diagnosed with AIS were prospectively examined. In this study, the patients were divided into two groups based on National Institutes of Health Stroke Scale score, including the severe (score ≥ 6, 36 patients) and mild (score < 6, 51 patients) groups. Quantitative analysis included diffusion-weighted imaging (DWI) diffusion restricted maximum area, three-dimensional arterial spin labeling (3D-ASL) low-perfusion zone maximum area, and 3D-ASL cerebral blood flow (CBF) values. For qualitative analysis, four-dimensional triggered angiography non-contrast enhanced (4D-TRANCE) was used to assess hemodynamics features with a 4-point grading system. Differences among multiple groups were evaluated by analysis of variance. Receiver operating characteristic (ROC) curves were generated to assess the predictive ability.

**Results:**

The severe group had 21 males and 15 females, while there were 27 males and 24 females in the mild group, with no statistically significant differences in age (64 ± 12 versus 63 ± 12 years, *P* > 0.05). Statistical differences were found between these two groups in 3D-ASL low-perfusion zone maximum area and 4D-TRANCE grade (*P* < 0.001). The area under the curve of the combined model of these two parameters was 0.950, with a sensitivity of 88.9% and a specificity of 92.2%.

**Conclusion:**

The combined application of 3D-ASL and 4D-TRANCE is of predictive significance in the clinical grading of acute ischemic cerebral infarction. It could provide a multiparametric and objective basis for further diagnosis and treatment selection.

## Introduction

Acute ischemic stroke (AIS) is the primary cause of adult disability worldwide and the second most common cause of death related to cerebrovascular disease (Huang et al., 2023). Ischemic cerebrovascular disease is mostly attributed to arterial stenosis or occlusion by atherosclerosis (Bonati et al., 2022). The etiological and pathophysiological processes of ischemic cerebrovascular disease are complex, with AIS exhibiting the highest disability rate. Impaired cerebral perfusion (CP) is one of the main pathological bases of ischemic cerebrovascular disease and is closely related to the clinical manifestations and severity of the disease (Thirugnanachandran et al., 2022). This is influenced not only by the degree of vascular stenosis but also by the efficacy of collateral circulation (Ginsberg, 2018). The assessments of recanalization state and collateral flow were associated with the National Institutes of Health Stroke Scale (NIHSS) score, information on local blood perfusion and collateral circulation is vital for the treatment of patients with AIS (Lee et al., 2022). Lateral tissue perfusion is an important determinant of tissue outcome in acute stroke, it maintains tissue viability before reperfusion and blood flow for longer periods of time. Researches demonstrated that patients with extensive collateral vessels tend to exhibit clinical outcome (Sasahara et al., 2024). Therefore, early assessment of collateral circulation and timely intervention are of vital significance for the clinical prognosis of stroke patients.

National Institutes of Health Stroke Scale score is a quantitative indicator of disease severity that is commonly employed as a surrogate endpoint in clinic (Yamal and Grotta, 2021). Patients are graded according to NIHSS score to guide clinical decision-making (Sasahara et al., 2024). It is a clinical tool that reliably and effectively assesses the severity of stroke in patients, and may assist doctors in predicting recovery, selecting treatment options, and developing subsequent rehabilitation plans. However, deficiencies exist in the clinical use of NIHSS score, e.g., facial paralysis, ataxia, dysarthria, and consciousness level. Differences in scoring between evaluators for the same patient may lead to inconsistent results, potentially resulting in variations in treatment options.

Traditional digital subtraction angiography (DSA) is the gold standard reference for the evaluation of intracranial vascular lesions. It has the capability to clearly depict the vascularity and blood flow direction. However, it is an invasive technique that requires contrast media and exposure to radiation (Wang M. et al., 2021). Magnetic resonance imaging (MRI) offers an attractive approach due to the lack of ionizing radiation and superior soft tissue contrast, especially for the brain tissue (Belani et al., 2020). Three-dimensional arterial spin labeling (3D-ASL) applies a rapid spin-echo sequence by employing water molecules in internal arterial blood as endogenous magnetic tracers, which can reflect microvascular perfusion levels with high image signal-to-noise ratio (SNR) (Jezzard et al., 2020). In addition, it is simple and non-invasive, making it suitable for measuring quantitative parameters (Sasahara et al., 2024). Moreover, the recognition of intracranial collateral vessels in 4D-TRANCE is highly consistent with DSA findings (Wang M. et al., 2021).

Arterial spin labeling-based four-dimensional triggered angiography non-contrast enhanced (4D-TRANCE) is an emerging non-invasive alternative to DSA, which magnetically labels the patient’s blood as an intrinsic contrast agent flowing through the brain (Suzuki et al., 2020). 4D-TRANCE captures the morphological and blood flow information of the cerebral vasculature (Phellan et al., 2020). Visualization of the perfusion regions of the main cerebral blood supply arteries hold the clinical value for diverse clinical applications at multiple time points (Wang M. et al., 2021). The information obtained from 4D-TRANCE can reflect the physiological blood flow condition (Suzuki et al., 2020). This approach has been used in several diseases, including moyamoya disease (MMD), arteriovenous malformation (AVM), and stroke (Wang M. et al., 2021). However, in the 4D-TRANCE studies on stroke patients, there are few studies combining clinical features for differentiating grades of ischemic stroke.

This study aimed to evaluate the potentials of 3D-ASL and 4D-TRANCE for providing dynamic data regarding intracranial vessels by visualizing and evaluating clinical grading in patients with acute ischemic cerebral infarction.

## Materials and methods

### Patients

**FIGURE 1:**
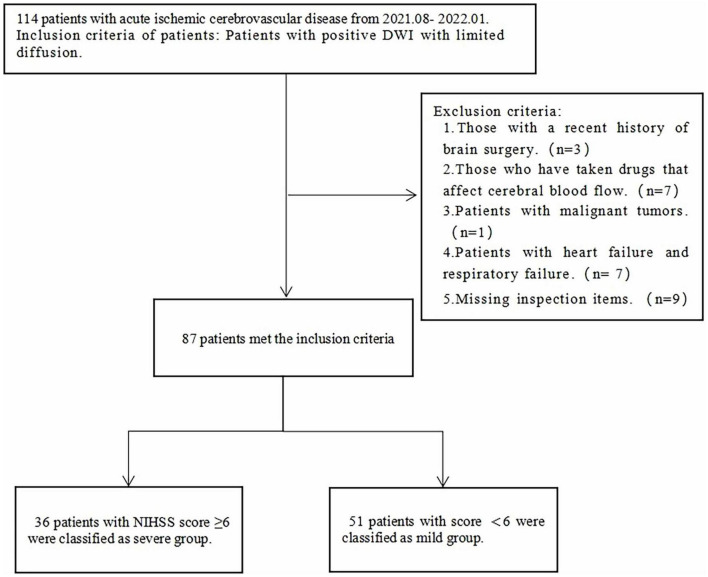
Inclusion criteria and study flowchart.

All patients were assessed for stroke severity using NIHSS score. Clinically, a NIHSS score ≥ 6 is usually considered a criterion for the detection of cases requiring intervention (Powers et al., 2018). Therefore, in this study, the patients were divided into two groups based on NIHSS score. The flowchart of inclusion and exclusion criteria are displayed in Figure 1.

### MRI acquisition and analysis

All participants were examined using a 3.0-T MR platform (Ingenia CX, Philips Healthcare, Netherlands) with a 32-channel head coil. The scanning protocol included diffusion-weighted imaging (DWI), T1-weighted imaging (T1WI), T2-weighted imaging (T2WI), T2-fluid attenuated inversion recovery (FLAIR), magnetic resonance angiography (MRA), 3D-ASL, and 4D-TRANCE sequences (Table 1). 3D-ASL was used with single delay acquisition, post-labeling delay (PLD) = 1,500 ms (Wang X. et al., 2021). ISP workstation (IntelliSpace Portal; Version 10.1; Philips Healthcare, Best, Netherlands) was used for post-processing by two radiologists with extensive experience (15 and 5 years of experience, respectively).

**TABLE 1:** Magnetic resonance imaging acquisition details.

Parameter	DWI	3D-ASL	4D-TRANCE	T1WI	T2WI	FLAIR	MRA
Sequence type	EPI	pCASL	TRANCE	TSE	TSE	IR	TOF
Scan time	00:12	04:42	06:40	00:56	00:24	01:12	01:58
TR	2,055	4,034	11	2,060	3,000	8,000	20
TE	83	12	5.8	20	90	120	3.5
FOV (mm^3^)	230 × 230 × 125	240 × 240 × 84	210 × 210 × 91	230 × 220 × 125	230 × 208 × 125	230 × 207 × 125	200 × 200 × 92
Scanning bit	TRA	TRA	TRA	TRA	TRA	TRA	TRA
Layer thickness (mm)	6	6	1.3	6	6	6	1.4
Acquisition matrix	128 × 101	64 × 60	172 × 150	256 × 212	288 × 143	208 × 150	304 × 173
Voxel (mm^3^)	1.8 × 2.5 × 6	3.75 × 3.75 × 6	1.22 × 1.22 × 1.3	0.9 × 1 × 6	0.8 × 1 × 6	1.11 × 1.37 × 6	0.66 × 1.15 × 1.4
SENSE	2.5	–	2.5	2	3	2.5	3

TR, repetition time; TE, echo time; FOV, field of view; SENSE, parallel acquisition.

### Quantitative analysis

Diffusion-weighted imaging analysis was processed as follows: the diffusion restricted area was selected, and two experienced radiologists (with 15 and 5 years of experience, respectively) manually delineated the largest slices. The 3D-ASL data were quantified as follows: firstly, in the 3D-ASL pseudo-color images, the slice with the largest hypoperfusion area was selected. Secondly, this largest hypoperfusion area was manually delineated as regions of interest (ROIs). Thirdly, the contralateral side of the brain were selected as the normal control. The cerebral blood flow (CBF) (ml/100 g/min) value was measured and calculated for each ROI (Jiang et al., 2022). The relative cerebral blood flow (rCBF) is defined as ischemic regional CBF/contralateral CBF. The rCBF is considered normal, if it is between 0.8 and 1.20. Values below 0.80 or above 1.20 are considered to indicate low perfusion and high perfusion, respectively (Wang X. et al., 2021).

### Qualitative analysis

Intracranial trunk and collateral vessels were evaluated with the 4D-TRANCE with a 4-point grading system and compared using MRA (Wang M. et al., 2021): 1, almost all blood vessels are visible; 2, more than half but not all blood vessels are visible; 3, less than half of blood vessels are visible; and 4, almost nothing is visible. The main and collateral vessels were shown on the dynamic image display in 4D, and the MIP image of MRA was used for comparative analysis. Static images of the whole brain were employed to analyze MRA data.

### Statistical analysis

SPSS version 26.0 (Armonk, NY: IBM Corp.) was utilized for statistical analysis. Normally distributed continuous variables were reported as mean ± SD, while those with non-normal distribution were presented as median (lower and upper quartiles). Differences in measurement data among multiple groups were evaluated by analysis of variance. Enumeration data were expressed as percentage, and the χ^2^ test was carried out for comparisons. Measurement and categorical data were compared between groups by the *t*-test and the Mann–Whitney *U* test, respectively. Univariate and multivariate logistic regression analysis was performed to determine significant independent predictive parameters. The DeLong test was used to compare the performance of the different prediction parameters. The combined model was also constructed which combined with significant differences in the factors. Receiver operating characteristic (ROC) curves were generated to assess the diagnostic ability. The diagnostic efficacy of single factor and multiple factor with statistical significance for the classification of patients with different NIHSS score was compared. *P* < 0.05 indicated a statistically significant difference. The intra-group correlation coefficient (ICC) was used to test the consistency between the two observers. The ICC value > 0.75 indicted good consistency; the ICC value between 0.40 and 0.75 a medium consistency, and the ICC value < 0.40 a poor consistency.

## Results

### Patients

A total of 87 patients were finally included in this study. There were 48 males and 39 females, aged 63 ± 12.25 years. The severe group had 21 males and 15 females, with aged 64 ± 12 years, while there were 27 males and 24 females in the mild group, aged 63 ± 12 years (Table 2). There were no significant differences in age, gender, and risk factors for acute cerebral infarction (hypertension, diabetes mellitus, smoking history, alcohol history, and hyperlipidemia) between the mild and severe groups (all *P* > 0.05) (Table 2).

**TABLE 2:** Baseline data in patients with high and low NIHSS scores.

Characteristics		NIHSS score		
	*N* = 87	<6 (*n* = 51)	≥6 (*n* = 36)	*P* value
Age (mean ± SD) (year)	63 ± 12	63 ± 12	64 ± 12	0.771
Sex (male/female)	48/39	27/24	21/15	0.907
BMI (kg/m^2^)	24 (22–26)	23 (21–25)	25 (22–26)	0.157
Time of onset (h)	9 (8–11)	9 (8–11)	9 (7–11)	0.709
Smoking history, no. (%)	32 (49.2)	20 (30.8)	12 (48)	0.878
Drinking history, no. (%)	16 (24.6)	10 (15.4)	6 (24)	0.929
History of hypertension, no. (%)	26 (40)	16 (24.6)	10 (40)	1.000
History of diabetes, no. (%)	16 (24.6)	10 (15.4)	6 (24)	0.929
Hyperlipidemia, no. (%)	19 (29.2)	10 (15.4)	9 (36)	0.351
MRA collateral circulation, no. (%)	27 (41.5)	18 (27.7)	9 (13.8)	<0.001
4D-TRANCE collateral circulation, no. (%)	53 (81.5)	37 (56.9)	16 (24.6)	<0.001
3D-ASL evaluation (CBF) (ml/100 g/min)	13 (5–22.5)	14.5 (8.25–23)	8 (3–20.5)	0.173
DWI Max area (cm^2^)	1.16 (0.54–3.54)	4.38 (1.9–9.89)	79.5 (28.5–120.5)	<0.001
3D-ASL Max area (cm^2^)	2.15 (0.23–9.31)	9.89 (5.66–39.4)	58.5 (7.5–210.5)	<0.001
4D-TRANCE grade	1 (1–2)	1 (1–1)	2 (2–3)	<0.001

ANOVA, analysis of variance; NIHSS, National Institutes of Health Stroke Scale; BMI, body mass index; CBF, cerebral blood flow.

### MRI data

Quantitative analysis showed that the maximum area of DWI (diffusion-limited zone) in the severe group was 79.5 (28.5–120.5) cm^2^, versus 4.38 (1.9–9.89) cm^2^ for the mild group. The maximum areas of 3D-ASL (low-perfusion zone) were 58.5 (7.5–210.5) cm^2^ and 9.89 (5.66–39.4) in the severe and mild groups, respectively. These values were statistically significant (*P* < 0.05, Table 2).

Qualitative analysis showed 4D-TRANCE grades 3 (3–4) and 2 (1.5–2) in the severe and mild groups, respectively (*P* < 0.05) (Table 2). There were no significant observer-to-observer differences using 4D-TRANCE and MRA for assessing intracranial artery vascular display (*P* > 0.05). In 45% of patients, there was a significant difference in the visualization of intracranial collateral vessels between 4D-TRANCE and MRA. A total of 34% of patients had a complete outflow delay, while 63% had inconsistent bilateral vascular flow velocities.

Logistic regression analyses showed that Further analysis of the binary regression showed that the maximum area of 4D-rotating grade and 3D-ASL were predictive factors (OR, 8.796; 2.602–29.739; *P* = 0.001 and OR, 1.002; 1.000–1.005; *P* = 0.031) for patient classification, while other factors were not statistically significant (*P* > 0.05).

**FIGURE 2:**
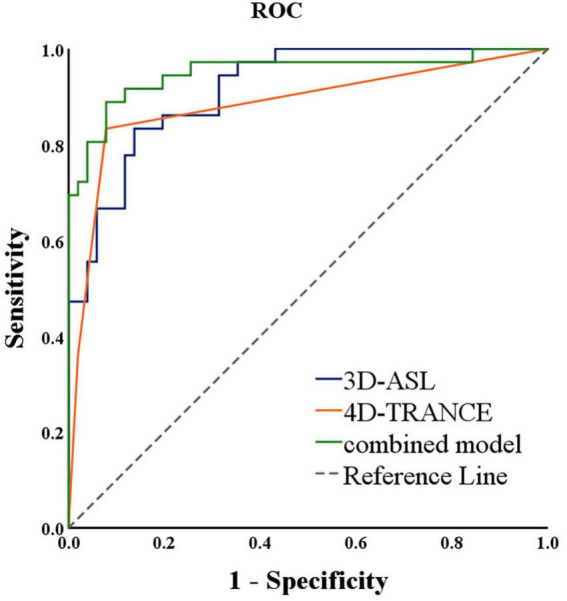
Receiver operating characteristic curve analysis of patients with multiple diagnostic methods.

Receiver operating characteristic curve analyses showed that the maximum area of 3D-ASL (low-perfusion zone) and 4D-TRANCE classification were independent predictive values (*P* < 0.05). The area under the curve (AUC) of 3D-ASL (low-perfusion zone) maximum area score was 0.916, with a sensitivity of 83.3% and a specificity of 86.3% (Table 3). The AUC of 4D-TRANCE grade was 0.884, with a sensitivity of 83.3% and a specificity of 92.2%. The AUC of the combined model (3D-ASL maximum area and 4D-TRANCE) was 0.950, with a sensitivity of 88.9% and a specificity of 92.2% (Figure 2).

**TABLE 3:** Results of ROC and binary logistic regression analyses of patients with multiple diagnostic methods.

Characteristic	AUC (95% CI)	Cut-off values	Specificity (%)	Sensitivity (%)	*P* value
4D-TRANCE grade	0.884 (0.802–0.965)	0.755	92.2	83.3	<0.001
3D-ASL Max area	0.916 (0.861–0.971)	0.696	86.3	83.3	<0.001
4D-TRANCE grade and 3D-ASL Max area combined model	0.950 (0.899–1.000)	0.811	92.2	88.9	<0.001

AUC, area under the curve; CI, confidence interval; ROC, receiver operating characteristic.

The results of the consistency analysis among observers are as follows: the ICC values of DWI Max area and 3D-ASL Max area manually drawn by observer 1 and observer 2 were 0.879 and 0.821, respectively. The ICC values of each parameter measured between the two observers were all >0.75, suggesting good agreement.

## Discussion

In this study, patients with acute cerebral infarction were divided into the severe and mild groups based on the clinical NIHSS score. The present study showed significant differences in the maximum area of 3D-ASL and 4D-TRANCE grade according to NIHSS score. The maximum area of 3D-ASL (low perfusion zone), maximum area of the 4D-TRANCE grade and the combined model had predictive values for the subgroups of severe and mild groups. The predictive ability of the combined model achieved significantly higher than those of the single models.

**FIGURE 3:**
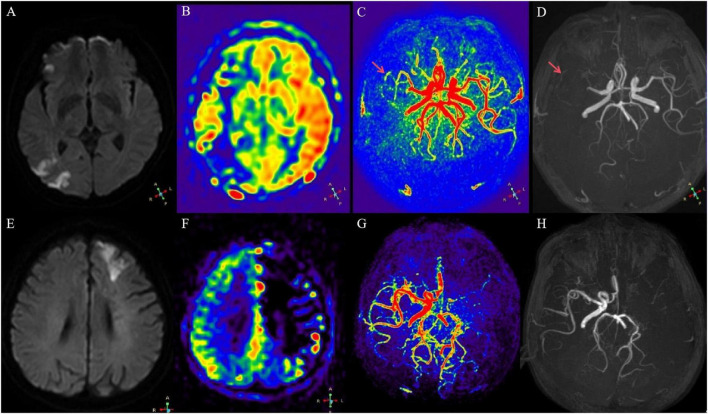
**(A–D)** A 48-year-old male with dizziness, right limb weakness, and sensory impairment. **(A)** Multiple and limited diffusion in the right frontal parietal lobe of DWI. **(B)** There was a large area of hypoperfusion around 3D-ASL lesions. **(C)** At the peak of vascular perfusion, 4D-TRANCE angiography showed that the M1 segment of the middle cerebral artery was occluded, and collateral vessels were visible (arrow). **(D)** TOF-MRA only showed occlusion of the M1 segment of the right middle cerebral artery (arrow). **(E–H)** A 49-year-old male with right lower limb weakness. **(E)** DWI showed limited left frontal lobe diffusion. **(F)** There was a large hypoperfusion area around 3D-ASL lesions. **(G)** At the peak of vascular perfusion, 4D-TRANCE angiography showed that the M1 segment of the middle cerebral artery was blocked, and no collateral vessels were found. **(H)** TOF-MRA showed occlusion of the M1 segment of left middle cerebral artery.

Diffusion-weighted imaging is considered the gold standard for defining the infarct core, with the infarct volume correlated with the severity of the neurological status (Wang et al., 2020). 3D-ASL is not only capable of effectively assessing and determining infarct size but also adept at evaluating hypoperfusion areas associated with neurological symptoms due to reduced blood supply (Shinohara et al., 2017). The mismatched area between the two images corresponds to the ischemic penumbra, which is located in the blood perfusion zone surrounding the cerebral infarction within the same vascular territory. Neurons in this region exhibit physiological and biochemical abnormalities caused by ischemia, leading to functional impairment. However, these neurons have not yet undergone irreversible damage. Timely restoration of adequate perfusion can normalize neuronal function, whereas further deterioration may result in progression to infarction and exacerbate brain injury (Ermine et al., 2021). Although collateral circulation possesses a certain degree of compensatory capacity, the extent of blood flow improvement varies. In this study, 3D-ASL imaging revealed that the area of reduced perfusion was significantly larger than the area of diffusion restriction observed on DWI in some patients, indicating the presence of an ischemic penumbra (Figure 3). These patients had higher clinical NIHSS scores, and the maximum area of 3D-ASL hypoperfusion was larger, while the maximum area of DWI diffusion restriction did not match the above two points. Therefore, robust collateral circulation can significantly improve the condition of insufficient blood supply (Figure 3). In addition, 3D-ASL imaging in some patients revealed no significant difference between the area of perfusion reduction and the area of DWI diffusion restriction. This indicates that the region of reduced blood supply largely overlapped with the cerebral infarction area, with no significant peri-infarct hypoperfusion observed, thereby limiting the opportunity for clinical salvage of the hypoperfused area. This suggests that ischemia associated with the affected vessels may persist in patients with inadequate collateral circulation, potentially leading to infarction over time. In addition, this study demonstrated that the extent of expansion around the infarct core lesion was relatively limited. Abundant collateral circulation appeared to mitigate the progression of the ischemic core (Figure 3), thereby further reducing the final infarct area and resulting in milder clinical symptoms, which is consistent with findings from previous study (Nam et al., 2020). In Figure 3C shows that during the peak of vascular perfusion, 4D-inferior flow angiography reveals an occlusion in segment M1 of the middle cerebral artery, with collateral vessels visible (arrows). Figure 3G also shows that during the peak of vascular perfusion, 4D-inferior flow angiography reveals an obstruction in segment M1 of the middle cerebral artery, but no collateral vessels are seen. The low perfusion area around the lesion in the corresponding 3D-ASL (Figure 3B) with collateral circulation is significantly smaller than the low perfusion area in the corresponding 3D-ASL (Figure 3F) without collateral circulation. Therefore, as a compensatory mechanism to maintain intracranial hemodynamic stability in stroke patients, the status of collateral circulation is of significant importance for the prognosis of ischemic stroke patients.

**FIGURE 4:**
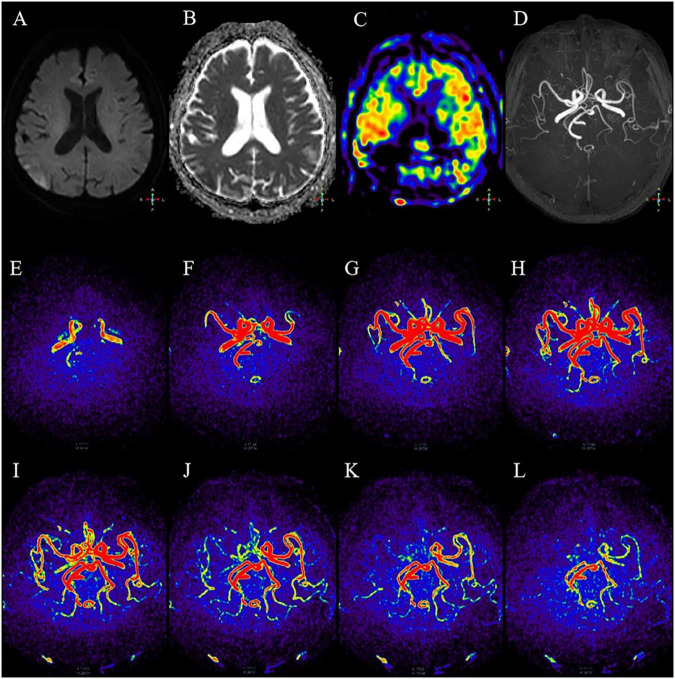
A 47-year-old male with dizziness, right limb weakness, and sensory impairment. **(A,B)** Local diffusion limitation in the right parietal lobe of DWI. **(C)** 3D-ASL showed large areas of underperfusion around the lesion. **(D)** No significant stenosis of the TOF-MRA cerebral artery. **(E–L)** According to the dynamic changes of blood flow in different periods of 4D-TRANCE, the right middle cerebral artery flowed faster than the left side. With an ischemic penumbra, bilateral blood flow velocity was discordant.

Four-dimensional triggered angiography non-contrast enhanced technology can detect stenosis of internal cerebral arteries, reflecting blood flow velocity and the deformation of collateral compensatory pathways (Phellan et al., 2020). It is also an effective non-invasive method to evaluate the patency of intracranial collateral vessels. In the present study, 4D-TRANCE assessment showed inconsistent bilateral vascular flow velocity in 63% of patients with AIS, which may indicate an inconsistent level of lateral arterial stenosis, resulting in an increased or decreased stenosis velocity, thus affecting blood supply in the infarcted area. Furthermore, 34% of cases had a complete outflow delay, and this proportion of the labeled flow still did not have a full 1.6 s labeling delay, indicating an overall slower flow rate and a perfusion delay (Figure 4). In Figures 4E–L show the process of blood flow dynamic changes in 4D-TRANCE at different periods, and the blood flow velocity of the middle cerebral artery on the right side is faster than that on the left side. There is an ischemic half dark band, and the blood flow velocity on both sides is inconsistent. Studies showed that cerebral vascular dilation may induce local hypoxia in the brain tissue (Phellan et al., 2020; Mikhail Kellawan et al., 2017; Kwater et al., 2020). Atherosclerosis risk factors, elevated blood fibrinogen levels, leukocytosis, mean arterial pressure, and increased cardiac output may be responsible for slow cerebral blood supply and ischemic symptoms (Kwater et al., 2020; Cinar et al., 2021; Lie et al., 2021).

The results of 4D-TRANCE can be replicated multiple times post-labeling to dynamically visualize the labeled blood flow entering the arterial tree (Suzuki et al., 2020). Additionally, 4D-TRANCE can demonstrate both the occluded feeding artery and the collateral circulation within the lesion area. Blood flow can be observed by the blood flow at different time points. The 4D-TRANCE can obtain macroscopic vessels, blood flow direction and microcirculation perfusion information simultaneously in one collection (Suzuki et al., 2020), which can provide more useful clinical information for patients with ischemic stroke compared with MRA. It can demonstrate the differences of bilateral flow between vessels and flow images. Furthermore, large vessel blood flow information helps to distinguish hyperperfusion from delayed residual arterial transport when post-treated perfusion images of vascular treatment for steno-occlusive disease are hyperintense (Suzuki et al., 2020). Our study also showed that 3D-ASL can effectively depict hypoperfusion lesions. 3D-ASL can reflect CP information and collateral circulation, and 4D-TRANCE can show the morphology and branches of arterial blood vessels and can show the direction of blood flow (Wang M. et al., 2021). In particular, it can be shown that the direction of collateral circulation flow is different from that of positive flow, and the combination of the two techniques can provide a more comprehensive and accurate assessment of AIS. The combination of these technologies can comprehensively evaluate the severity of acute ischemic cerebral infarction, brain parenchyma perfusion, and vascular morphology. This integration preliminarily elucidates blood flow dynamics, providing more comprehensive diagnostic information for clinical applications and an essential basis for subsequent interventional treatments and thrombolytic therapies.

This study has some limitations. First, 4D-TRANCE lacked quantitative results. Further research is needed with technological advancement for its application in improving diagnostic accuracy. Second, the study was a single-center study with a relatively small sample size, which might limit the generalizability of the results and lead to region-specific findings. In the future, multi-center studies are planned to conduct sensitivity analyses of the evaluation system and verify its robustness.

## Conclusion

Mild and severe groups showed significant differences in imaging examination results, with the severe group’s data notably higher than that of the mild group. Arterial blood flow velocity may be related to the severity of acute stroke, data includes: 3D-ASL (low-perfusion zone) maximum area and 4D-TRANCE grade. Both 3D-ASL (low-perfusion zone) maximum area and 4D-TRANCE grade models had predictive power for both groups, and the combined use of these two examination methods significantly improved sensitivity and specificity. In conclusion, the combined application of 3D-ASL and 4D-TRANCE has predictive significance for grading acute ischemic cerebral infarction. This combined approach has potential to provide reference for clinical decision-making of further therapies.

## Data Availability

The raw data supporting the conclusions of this article will be made available by the authors, without undue reservation.
